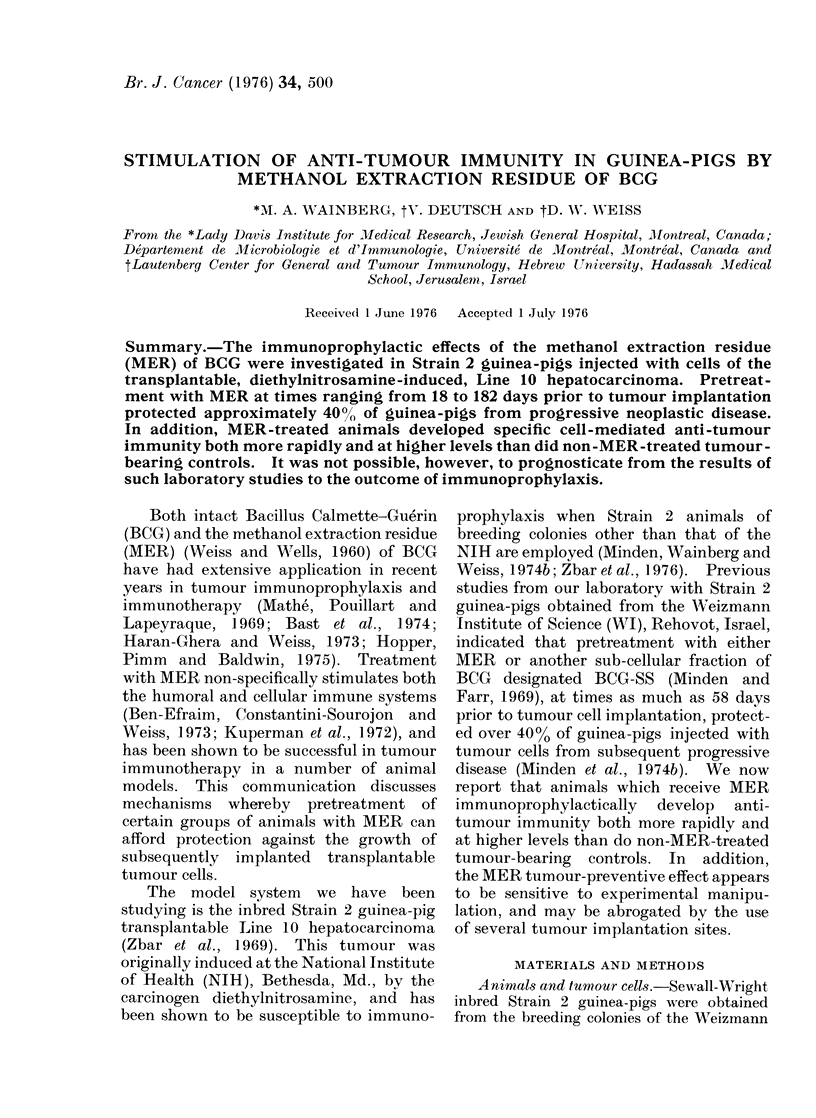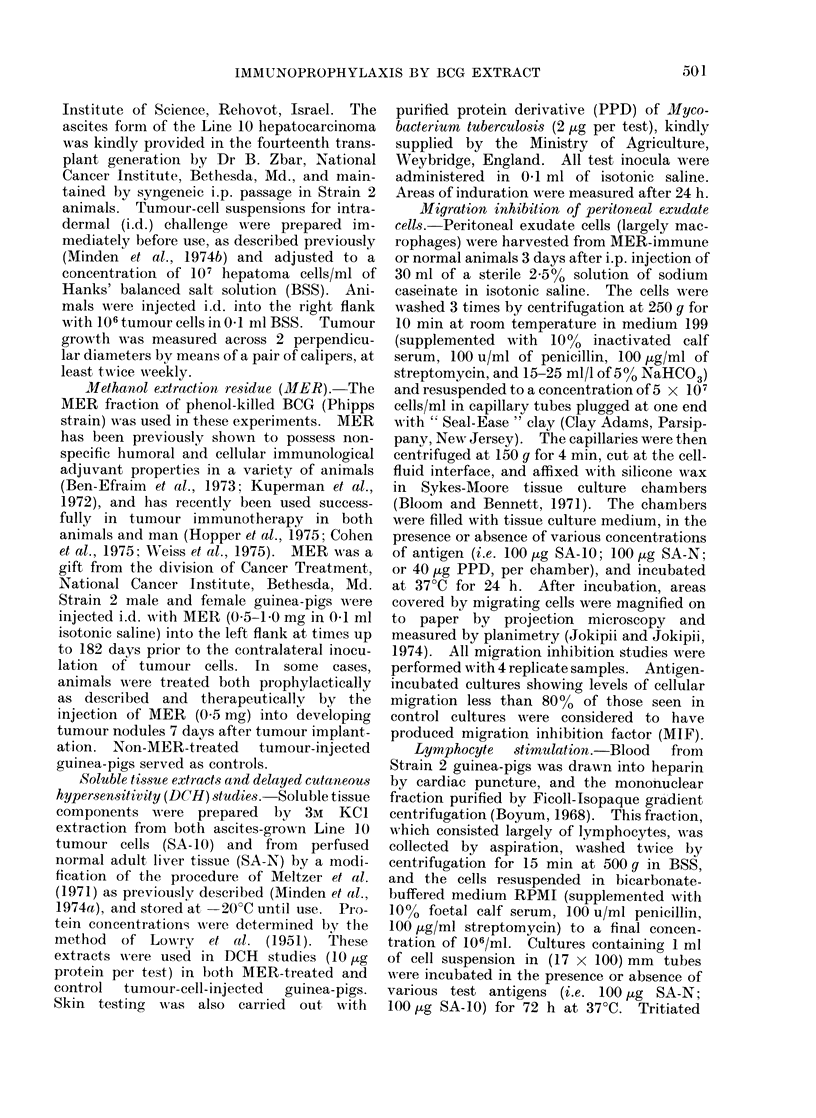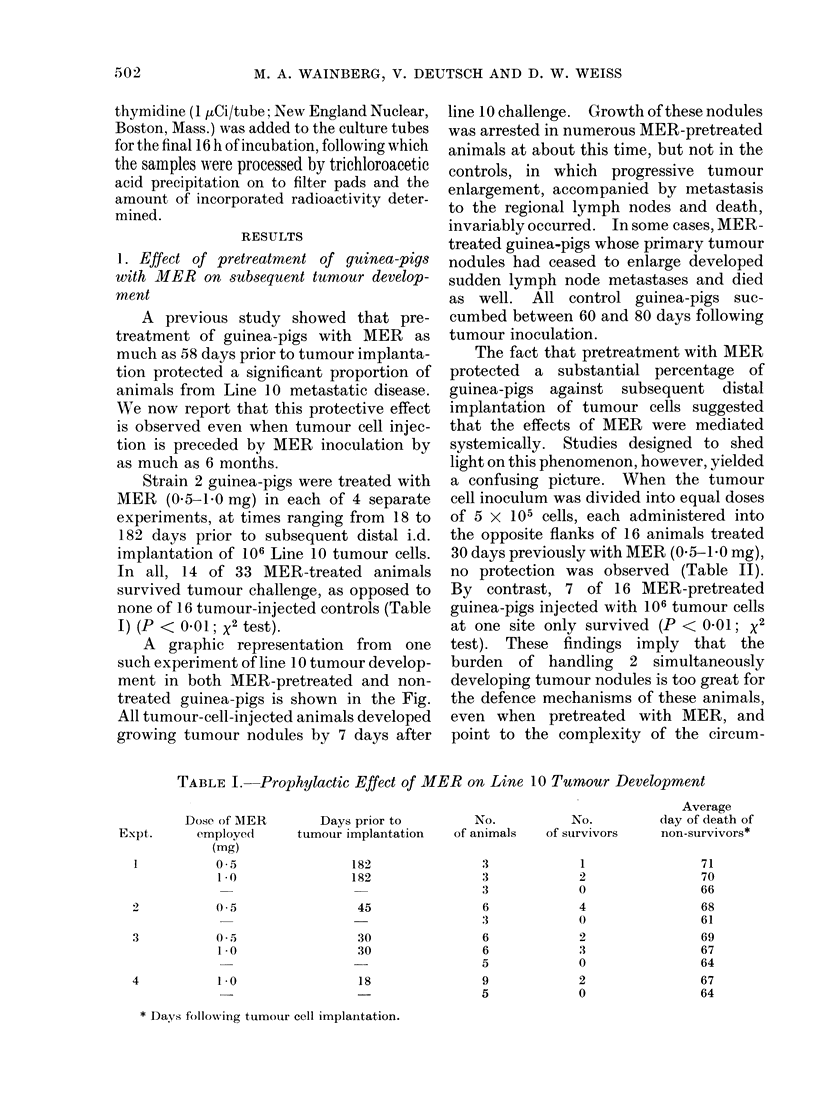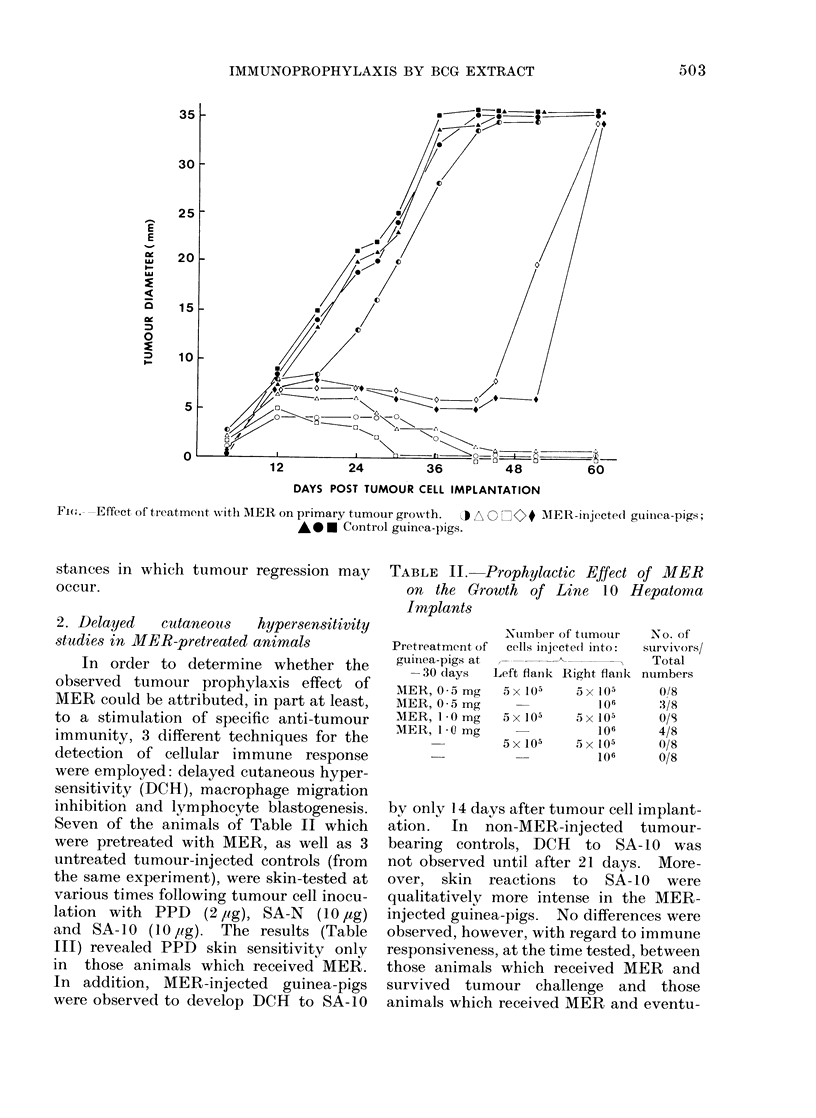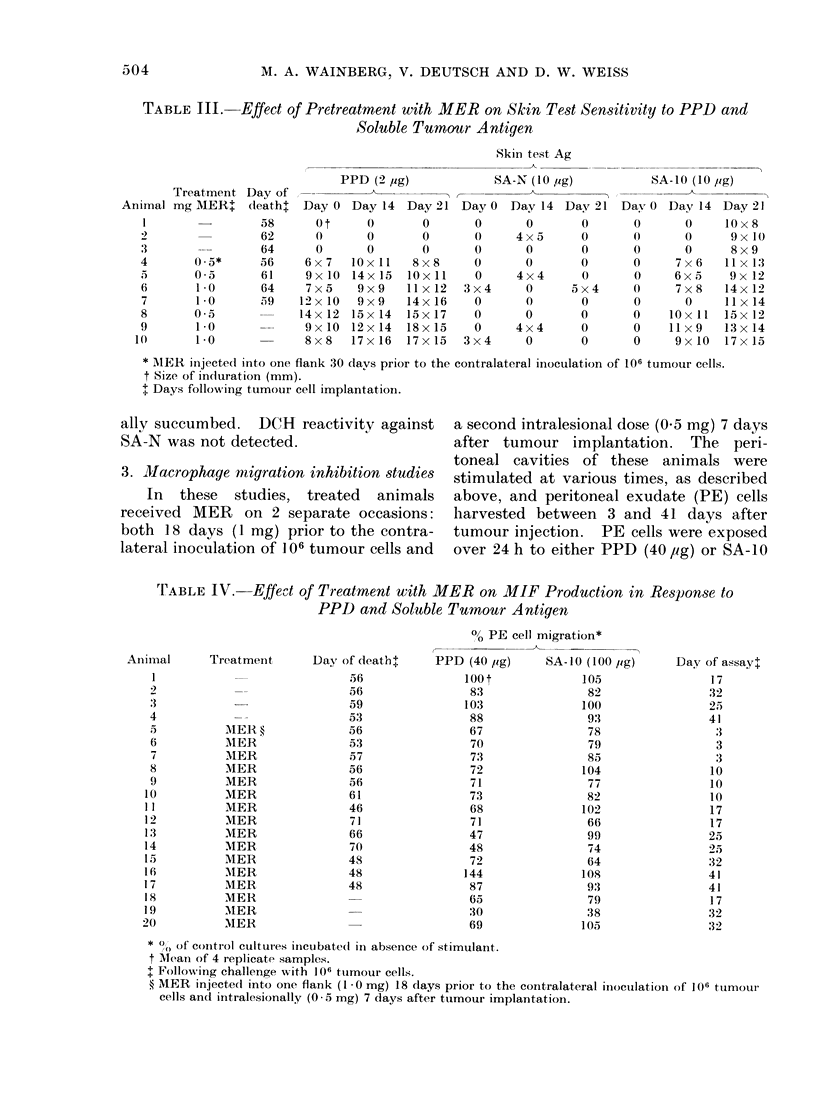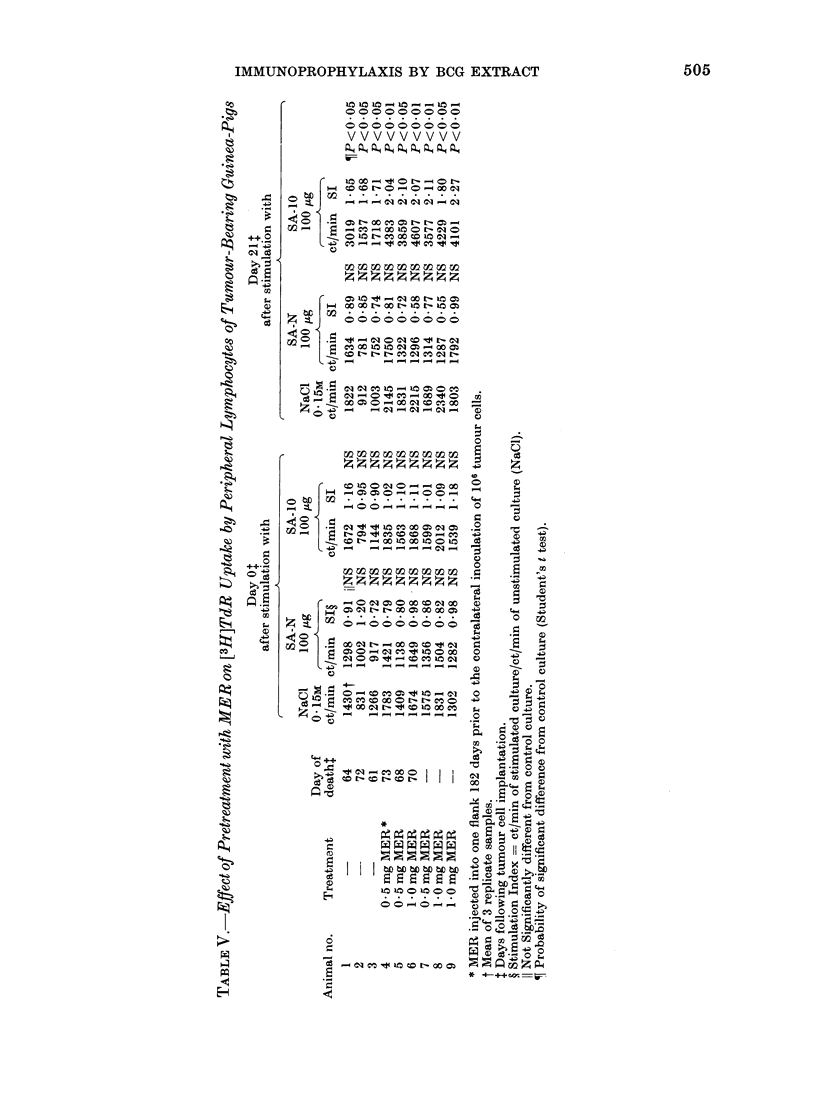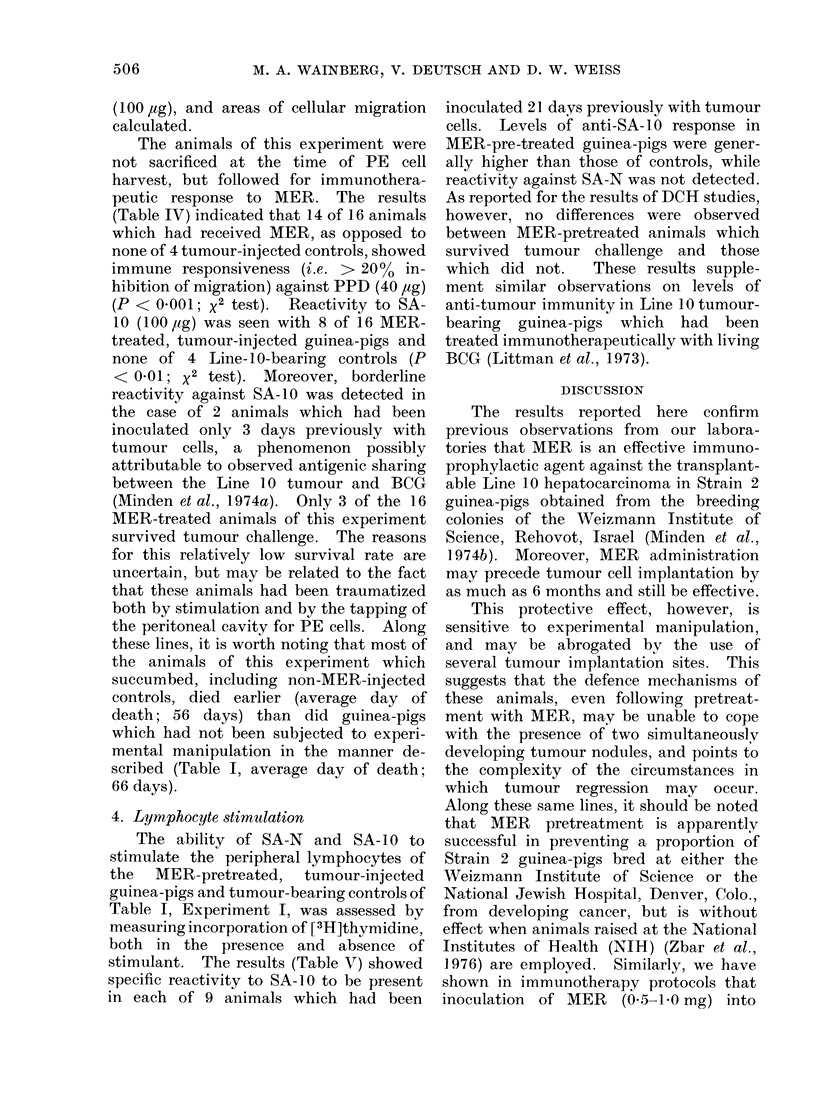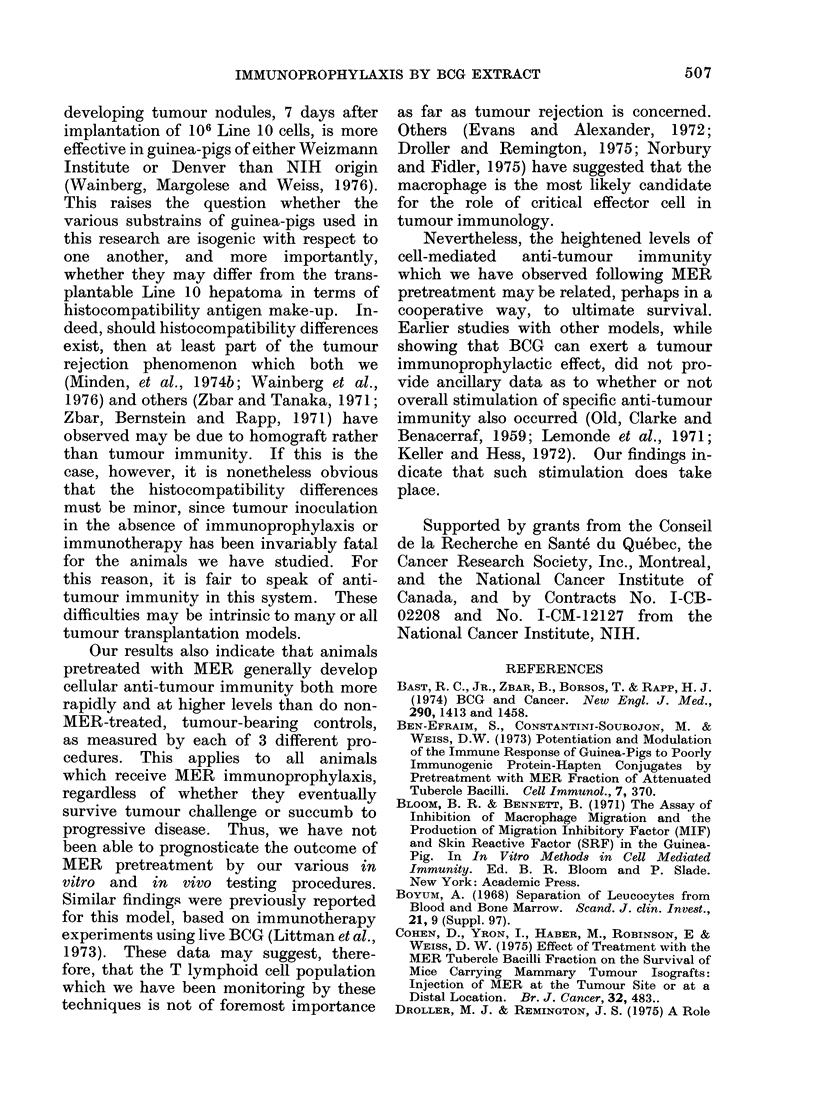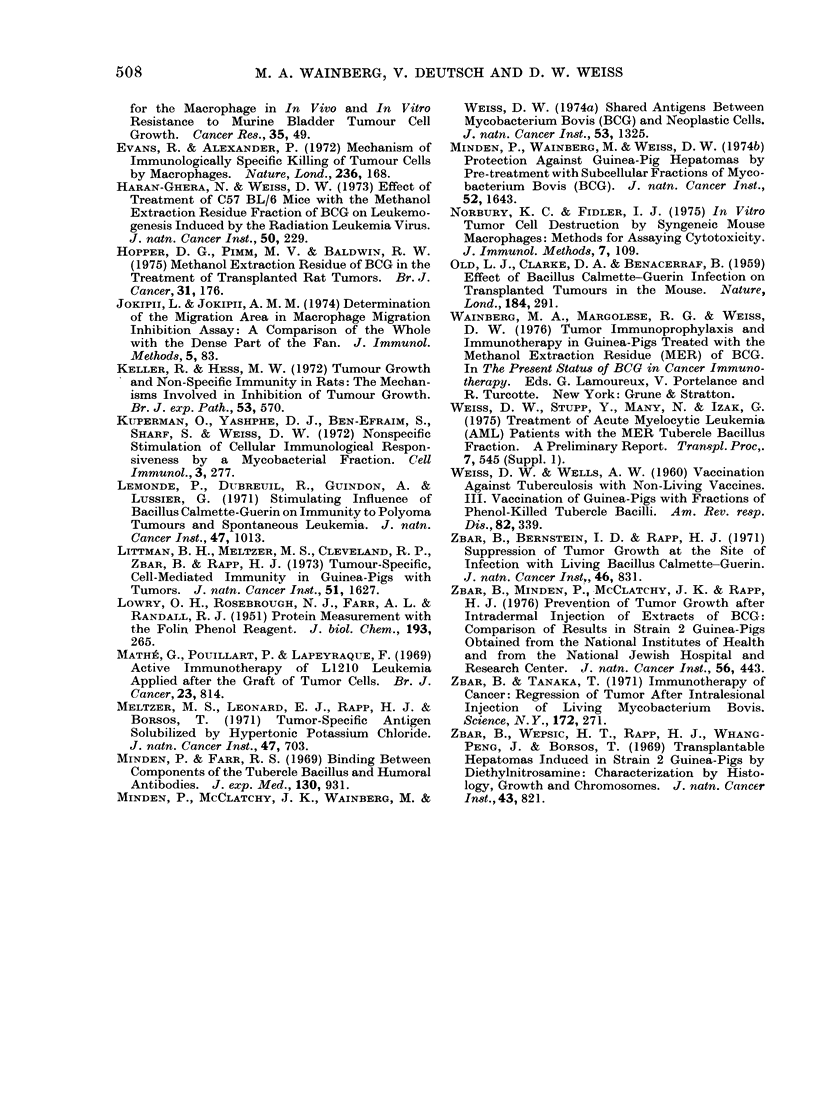# Stimulation of anti-tumour immunity in guinea-pigs by methanol extraction residue of BCG.

**DOI:** 10.1038/bjc.1976.204

**Published:** 1976-11

**Authors:** M. A. Wainberg, V. Deutsch, D. W. Weiss

## Abstract

The immunoprophylactic effects of the methanol extraction residue (MER) of BCG were investigated in Strain 2 guinea-pigs injected with cells of the transplantable, diethylnitrosamine-induced, Line 10 hepatocarcinoma. Pretreatment with MER at times ranging from 18 to 182 days prior to tumour implantation protected approximately 40% of guinea-pigs from progressive neoplastic disease. In addition, MER-treated animals developed specific cell-mediated anti-tumour immunity both more rapidly and at higher levels than did non-MER-treated tumour-bearing controls. It was not possible, however, to prognosticate from the results of such laboratory studies to the outcome of immunoprophylaxis.


*Br. J. Cancer* (1976) **34**, 500

### STIMULATION OF ANTI-TUMOUR IMMUNITY IN GUINEA-PIGS BY METHANOL EXTRACTION RESIDUE OF BCG

*M. A. WAINBERG, †V. DEUTSCH AND †D. W. WEISS

*From the *Lady Davis Institute for Medical Research, Jewish General Hospital, Montreal, Canada; Département de Microbiologie et d'Immunologie, Université de Montréal, Montréal, Canada and †Lautenberg Center for General and Tumour Immunology, Hebrew University, Hadassah Medical School, Jerusalem, Israel*

Received 1 June 1976 Accepted 1 July 1976

**Summary.—The immunoprophylactic effects of the methanol extraction residue (MER) of BCG were investigated in Strain 2 guinea-pigs injected with cells of the transplantable, diethylnitrosamine-induced, Line 10 hepatocarcinoma. Pretreatment with MER at times ranging from 18 to 182 days prior to tumour implantation protected approximately 40% of guinea-pigs from progressive neoplastic disease. In addition, MER-treated animals developed specific cell-mediated anti-tumour immunity both more rapidly and at higher levels than did non-MER-treated tumour-bearing controls. It was not possible, however, to prognosticate from the results of such laboratory studies to the outcome of immunoprophylaxis.**

Both intact Bacillus Calmette–Guérin (BCG) and the methanol extraction residue (MER) (Weiss and Wells, 1960) of BCG have had extensive application in recent years in tumour immunoprophylaxis and immunotherapy (Mathé, Pouillart and Lapeyraque, 1969; Bast *et al.*, 1974; Haran-Ghera and Weiss, 1973; Hopper, Pimm and Baldwin, 1975). Treatment with MER non-specifically stimulates both the humoral and cellular immune systems (Ben-Efraim, Constantini-Sourojon and Weiss, 1973; Kuperman *et al.*, 1972), and has been shown to be successful in tumour immunotherapy in a number of animal models. This communication discusses mechanisms whereby pretreatment of certain groups of animals with MER can afford protection against the growth of subsequently implanted transplantable tumour cells.

The model system we have been studying is the inbred Strain 2 guinea-pig transplantable Line 10 hepatocarcinoma (Zbar *et al.*, 1969). This tumour was originally induced at the National Institute of Health (NIH), Bethesda, Md., by the carcinogen diethylnitrosamine, and has been shown to be susceptible to immunoprophylaxis when Strain 2 animals of breeding colonies other than that of the NIH are employed (Minden, Wainberg and Weiss, 1974*b*; Zbar *et al.*, 1976). Previous studies from our laboratory with Strain 2 guinea-pigs obtained from the Weizmann Institute of Science (WI), Rehovot, Israel, indicated that pretreatment with either MER or another sub-cellular fraction of BCG designated BCG-SS (Minden and Farr, 1969), at times as much as 58 days prior to tumour cell implantation, protected over 40% of guinea-pigs injected with tumour cells from subsequent progressive disease (Minden *et al.*, 1974*b*). We now report that animals which receive MER immunoprophylactically develop anti-tumour immunity both more rapidly and at higher levels than do non-MER-treated tumour-bearing controls. In addition, the MER tumour-preventive effect appears to be sensitive to experimental manipulation, and may be abrogated by the use of several tumour implantation sites.

### MATERIALS AND METHODS

*Animals and tumour cells.*—Sewall-Wright inbred Strain 2 guinea-pigs were obtained from the breeding colonies of the Weizmann

Institute of Science, Rehovot, Israel. The ascites form of the Line 10 hepatocarcinoma was kindly provided in the fourteenth transplant generation by Dr B. Zbar, National Cancer Institute, Bethesda, Md., and maintained by syngeneic i.p. passage in Strain 2 animals. Tumour-cell suspensions for intradermal (i.d.) challenge were prepared immediately before use, as described previously (Minden *et al.*, 1974*b*) and adjusted to a concentration of $10^7$ hepatoma cells/ml of Hanks' balanced salt solution (BSS). Animals were injected i.d. into the right flank with $10^6$ tumour cells in 0·1 ml BSS. Tumour growth was measured across 2 perpendicular diameters by means of a pair of calipers, at least twice weekly.

*Methanol extraction residue* (*MER*).—The MER fraction of phenol-killed BCG (Phipps strain) was used in these experiments. MER has been previously shown to possess non-specific humoral and cellular immunological adjuvant properties in a variety of animals (Ben-Efraim *et al.*, 1973; Kuperman *et al.*, 1972), and has recently been used successfully in tumour immunotherapy in both animals and man (Hopper *et al.*, 1975; Cohen *et al.*, 1975; Weiss *et al.*, 1975). MER was a gift from the division of Cancer Treatment, National Cancer Institute, Bethesda, Md. Strain 2 male and female guinea-pigs were injected i.d. with MER (0·5–1·0 mg in 0·1 ml isotonic saline) into the left flank at times up to 182 days prior to the contralateral inoculation of tumour cells. In some cases, animals were treated both prophylactically as described and therapeutically by the injection of MER (0·5 mg) into developing tumour nodules 7 days after tumour implantation. Non-MER-treated tumour-injected guinea-pigs served as controls.

*Soluble tissue extracts and delayed cutaneous hypersensitivity* (*DCH*) *studies*.—Soluble tissue components were prepared by 3M KCl extraction from both ascites-grown Line 10 tumour cells (SA-10) and from perfused normal adult liver tissue (SA-N) by a modification of the procedure of Meltzer *et al.* (1971) as previously described (Minden *et al.*, 1974*a*), and stored at −20°C until use. Protein concentrations were determined by the method of Lowry *et al.* (1951). These extracts were used in DCH studies (10 μg protein per test) in both MER-treated and control tumour-cell-injected guinea-pigs. Skin testing was also carried out with purified protein derivative (PPD) of *Mycobacterium tuberculosis* (2 μg per test), kindly supplied by the Ministry of Agriculture, Weybridge, England. All test inocula were administered in 0·1 ml of isotonic saline. Areas of induration were measured after 24 h.

*Migration inhibition of peritoneal exudate cells*.—Peritoneal exudate cells (largely macrophages) were harvested from MER-immune or normal animals 3 days after i.p. injection of 30 ml of a sterile 2·5% solution of sodium caseinate in isotonic saline. The cells were washed 3 times by centrifugation at 250 *g* for 10 min at room temperature in medium 199 (supplemented with 10% inactivated calf serum, 100 u/ml of penicillin, 100 μg/ml of streptomycin, and 15–25 ml/l of 5% $NaHCO_3$) and resuspended to a concentration of $5 \times 10^7$ cells/ml in capillary tubes plugged at one end with "Seal-Ease" clay (Clay Adams, Parsippany, New Jersey). The capillaries were then centrifuged at 150 *g* for 4 min, cut at the cell-fluid interface, and affixed with silicone wax in Sykes-Moore tissue culture chambers (Bloom and Bennett, 1971). The chambers were filled with tissue culture medium, in the presence or absence of various concentrations of antigen (*i.e.* 100 μg SA-10; 100 μg SA-N; or 40 μg PPD, per chamber), and incubated at 37°C for 24 h. After incubation, areas covered by migrating cells were magnified on to paper by projection microscopy and measured by planimetry (Jokipii and Jokipii, 1974). All migration inhibition studies were performed with 4 replicate samples. Antigen-incubated cultures showing levels of cellular migration less than 80% of those seen in control cultures were considered to have produced migration inhibition factor (MIF).

*Lymphocyte stimulation*.—Blood from Strain 2 guinea-pigs was drawn into heparin by cardiac puncture, and the mononuclear fraction purified by Ficoll-Isopaque gradient centrifugation (Boyum, 1968). This fraction, which consisted largely of lymphocytes, was collected by aspiration, washed twice by centrifugation for 15 min at 500 *g* in BSS, and the cells resuspended in bicarbonate-buffered medium RPMI (supplemented with 10% foetal calf serum, 100 u/ml penicillin, 100 μg/ml streptomycin) to a final concentration of $10^6$/ml. Cultures containing 1 ml of cell suspension in (17 × 100) mm tubes were incubated in the presence or absence of various test antigens (*i.e.* 100 μg SA-N; 100 μg SA-10) for 72 h at 37°C. Tritiated thymidine (1 $\mu$Ci/tube; New England Nuclear, Boston, Mass.) was added to the culture tubes for the final 16 h of incubation, following which the samples were processed by trichloroacetic acid precipitation on to filter pads and the amount of incorporated radioactivity determined.

### RESULTS

### 1. *Effect of pretreatment of guinea-pigs with MER on subsequent tumour development*

A previous study showed that pretreatment of guinea-pigs with MER as much as 58 days prior to tumour implantation protected a significant proportion of animals from Line 10 metastatic disease. We now report that this protective effect is observed even when tumour cell injection is preceded by MER inoculation by as much as 6 months.

Strain 2 guinea-pigs were treated with MER (0·5–1·0 mg) in each of 4 separate experiments, at times ranging from 18 to 182 days prior to subsequent distal i.d. implantation of $10^6$ Line 10 tumour cells. In all, 14 of 33 MER-treated animals survived tumour challenge, as opposed to none of 16 tumour-injected controls (Table I) ($P < 0{\cdot}01$; $\chi^2$ test).

A graphic representation from one such experiment of line 10 tumour development in both MER-pretreated and non-treated guinea-pigs is shown in the Fig. All tumour-cell-injected animals developed growing tumour nodules by 7 days after line 10 challenge. Growth of these nodules was arrested in numerous MER-pretreated animals at about this time, but not in the controls, in which progressive tumour enlargement, accompanied by metastasis to the regional lymph nodes and death, invariably occurred. In some cases, MER-treated guinea-pigs whose primary tumour nodules had ceased to enlarge developed sudden lymph node metastases and died as well. All control guinea-pigs succumbed between 60 and 80 days following tumour inoculation.

The fact that pretreatment with MER protected a substantial percentage of guinea-pigs against subsequent distal implantation of tumour cells suggested that the effects of MER were mediated systemically. Studies designed to shed light on this phenomenon, however, yielded a confusing picture. When the tumour cell inoculum was divided into equal doses of $5 \times 10^5$ cells, each administered into the opposite flanks of 16 animals treated 30 days previously with MER (0·5–1·0 mg), no protection was observed (Table II). By contrast, 7 of 16 MER-pretreated guinea-pigs injected with $10^6$ tumour cells at one site only survived ($P < 0{\cdot}01$; $\chi^2$ test). These findings imply that the burden of handling 2 simultaneously developing tumour nodules is too great for the defence mechanisms of these animals, even when pretreated with MER, and point to the complexity of the circum-

TABLE I.—*Prophylactic Effect of MER on Line 10 Tumour Development*

| Expt. | Dose of MER employed (mg) | Days prior to tumour implantation | No. of animals | No. of survivors | Average day of death of non-survivors* |
|---|---|---|---|---|---|
| 1 | 0·5 | 182 | 3 | 1 | 71 |
| | 1·0 | 182 | 3 | 2 | 70 |
| | — | — | 3 | 0 | 66 |
| 2 | 0·5 | 45 | 6 | 4 | 68 |
| | — | — | 3 | 0 | 61 |
| 3 | 0·5 | 30 | 6 | 2 | 69 |
| | 1·0 | 30 | 6 | 3 | 67 |
| | — | — | 5 | 0 | 64 |
| 4 | 1·0 | 18 | 9 | 2 | 67 |
| | — | — | 5 | 0 | 64 |

* Days following tumour cell implantation.

FIG.—Effect of treatment with MER on primary tumour growth. ◐△○□◇♦ MER-injected guinea-pigs; ▲●■ Control guinea-pigs.

stances in which tumour regression may occur.

2. *Delayed cutaneous hypersensitivity studies in MER-pretreated animals*

In order to determine whether the observed tumour prophylaxis effect of MER could be attributed, in part at least, to a stimulation of specific anti-tumour immunity, 3 different techniques for the detection of cellular immune response were employed: delayed cutaneous hypersensitivity (DCH), macrophage migration inhibition and lymphocyte blastogenesis. Seven of the animals of Table II which were pretreated with MER, as well as 3 untreated tumour-injected controls (from the same experiment), were skin-tested at various times following tumour cell inoculation with PPD (2 μg), SA-N (10 μg) and SA-10 (10 μg). The results (Table III) revealed PPD skin sensitivity only in those animals which received MER. In addition, MER-injected guinea-pigs were observed to develop DCH to SA-10 by only 14 days after tumour cell implantation. In non-MER-injected tumour-bearing controls, DCH to SA-10 was not observed until after 21 days. Moreover, skin reactions to SA-10 were qualitatively more intense in the MER-injected guinea-pigs. No differences were observed, however, with regard to immune responsiveness, at the time tested, between those animals which received MER and survived tumour challenge and those animals which received MER and eventu-

TABLE II.—*Prophylactic Effect of MER on the Growth of Line 10 Hepatoma Implants*

| Pretreatment of guinea-pigs at −30 days | Number of tumour cells injected into: Left flank | Right flank | No. of survivors/ Total numbers |
|---|---|---|---|
| MER, 0·5 mg | $5\times10^5$ | $5\times10^5$ | 0/8 |
| MER, 0·5 mg | — | $10^6$ | 3/8 |
| MER, 1·0 mg | $5\times10^5$ | $5\times10^5$ | 0/8 |
| MER, 1·0 mg | — | $10^6$ | 4/8 |
| — | $5\times10^5$ | $5\times10^5$ | 0/8 |
| — | — | $10^6$ | 0/8 |

TABLE III.—*Effect of Pretreatment with MER on Skin Test Sensitivity to PPD and Soluble Tumour Antigen*

| | | | Skin test Ag | | | | | | | | |
|---|---|---|---|---|---|---|---|---|---|---|---|
| | | | PPD (2 μg) | | | SA-N (10 μg) | | | SA-10 (10 μg) | | |
| Animal | Treatment mg MER‡ | Day of death‡ | Day 0 | Day 14 | Day 21 | Day 0 | Day 14 | Day 21 | Day 0 | Day 14 | Day 21 |
| 1 | — | 58 | 0† | 0 | 0 | 0 | 0 | 0 | 0 | 0 | 10×8 |
| 2 | — | 62 | 0 | 0 | 0 | 0 | 4×5 | 0 | 0 | 0 | 9×10 |
| 3 | — | 64 | 0 | 0 | 0 | 0 | 0 | 0 | 0 | 0 | 8×9 |
| 4 | 0·5* | 56 | 6×7 | 10×11 | 8×8 | 0 | 0 | 0 | 0 | 7×6 | 11×13 |
| 5 | 0·5 | 61 | 9×10 | 14×15 | 10×11 | 0 | 4×4 | 0 | 0 | 6×5 | 9×12 |
| 6 | 1·0 | 64 | 7×5 | 9×9 | 11×12 | 3×4 | 0 | 5×4 | 0 | 7×8 | 14×12 |
| 7 | 1·0 | 59 | 12×10 | 9×9 | 14×16 | 0 | 0 | 0 | 0 | 0 | 11×14 |
| 8 | 0·5 | — | 14×12 | 15×14 | 15×17 | 0 | 0 | 0 | 0 | 10×11 | 15×12 |
| 9 | 1·0 | — | 9×10 | 12×14 | 18×15 | 0 | 4×4 | 0 | 0 | 11×9 | 13×14 |
| 10 | 1·0 | — | 8×8 | 17×16 | 17×15 | 3×4 | 0 | 0 | 0 | 9×10 | 17×15 |

* MER injected into one flank 30 days prior to the contralateral inoculation of $10^6$ tumour cells.
† Size of induration (mm).
‡ Days following tumour cell implantation.

ally succumbed. DCH reactivity against SA-N was not detected.

3. *Macrophage migration inhibition studies*

In these studies, treated animals received MER on 2 separate occasions: both 18 days (1 mg) prior to the contralateral inoculation of $10^6$ tumour cells and a second intralesional dose (0·5 mg) 7 days after tumour implantation. The peritoneal cavities of these animals were stimulated at various times, as described above, and peritoneal exudate (PE) cells harvested between 3 and 41 days after tumour injection. PE cells were exposed over 24 h to either PPD (40 μg) or SA-10

TABLE IV.—*Effect of Treatment with MER on MIF Production in Response to PPD and Soluble Tumour Antigen*

| | | | % PE cell migration* | | |
|---|---|---|---|---|---|
| Animal | Treatment | Day of death‡ | PPD (40 μg) | SA-10 (100 μg) | Day of assay‡ |
| 1 | — | 56 | 100† | 105 | 17 |
| 2 | — | 56 | 83 | 82 | 32 |
| 3 | — | 59 | 103 | 100 | 25 |
| 4 | — | 53 | 88 | 93 | 41 |
| 5 | MER § | 56 | 67 | 78 | 3 |
| 6 | MER | 53 | 70 | 79 | 3 |
| 7 | MER | 57 | 73 | 85 | 3 |
| 8 | MER | 56 | 72 | 104 | 10 |
| 9 | MER | 56 | 71 | 77 | 10 |
| 10 | MER | 61 | 73 | 82 | 10 |
| 11 | MER | 46 | 68 | 102 | 17 |
| 12 | MER | 71 | 71 | 66 | 17 |
| 13 | MER | 66 | 47 | 99 | 25 |
| 14 | MER | 70 | 48 | 74 | 25 |
| 15 | MER | 48 | 72 | 64 | 32 |
| 16 | MER | 48 | 144 | 108 | 41 |
| 17 | MER | 48 | 87 | 93 | 41 |
| 18 | MER | — | 65 | 79 | 17 |
| 19 | MER | — | 30 | 38 | 32 |
| 20 | MER | — | 69 | 105 | 32 |

* % of control cultures incubated in absence of stimulant.
† Mean of 4 replicate samples.
‡ Following challenge with $10^6$ tumour cells.
§ MER injected into one flank (1·0 mg) 18 days prior to the contralateral inoculation of $10^6$ tumour cells and intralesionally (0·5 mg) 7 days after tumour implantation.

TABLE V.—*Effect of Pretreatment with MER on [$^3H$]TdR Uptake by Peripheral Lymphocytes of Tumour-Bearing Guinea-Pigs*

| | | | Day 0‡ after stimulation with | | | | | | | Day 21‡ after stimulation with | | | | | | |
|---|---|---|---|---|---|---|---|---|---|---|---|---|---|---|---|---|
| | | | NaCl 0·15M | SA-N 100 μg | | | SA-10 100 μg | | | NaCl 0·15M | SA-N 100 μg | | | SA-10 100 μg | | |
| Animal no. | Treatment | Day of death‡ | ct/min | ct/min | SI§ | | ct/min | SI | | ct/min | ct/min | SI | | ct/min | SI | |
| 1 | — | 64 | 1430† | 1298 | 0·91 | ‖NS | 1672 | 1·16 | NS | 1822 | 1634 | 0·89 | NS | 3019 | 1·65 | ¶$P<0·05$ |
| 2 | — | 72 | 831 | 1002 | 1·20 | NS | 794 | 0·95 | NS | 912 | 781 | 0·85 | NS | 1537 | 1·68 | $P<0·05$ |
| 3 | — | 61 | 1266 | 917 | 0·72 | NS | 1144 | 0·90 | NS | 1003 | 752 | 0·74 | NS | 1718 | 1·71 | $P<0·05$ |
| 4 | 0·5 mg MER* | 73 | 1783 | 1421 | 0·79 | NS | 1835 | 1·02 | NS | 2145 | 1750 | 0·81 | NS | 4383 | 2·04 | $P<0·01$ |
| 5 | 0·5 mg MER | 68 | 1409 | 1138 | 0·80 | NS | 1563 | 1·10 | NS | 1831 | 1322 | 0·72 | NS | 3859 | 2·10 | $P<0·05$ |
| 6 | 1·0 mg MER | 70 | 1674 | 1649 | 0·98 | NS | 1868 | 1·11 | NS | 2215 | 1296 | 0·58 | NS | 4607 | 2·07 | $P<0·01$ |
| 7 | 0·5 mg MER | — | 1575 | 1356 | 0·86 | NS | 1599 | 1·01 | NS | 1689 | 1314 | 0·77 | NS | 3577 | 2·11 | $P<0·01$ |
| 8 | 1·0 mg MER | — | 1831 | 1504 | 0·82 | NS | 2012 | 1·09 | NS | 2340 | 1287 | 0·55 | NS | 4229 | 1·80 | $P<0·05$ |
| 9 | 1·0 mg MER | — | 1302 | 1282 | 0·98 | NS | 1539 | 1·18 | NS | 1803 | 1792 | 0·99 | NS | 4101 | 2·27 | $P<0·01$ |

* MER injected into one flank 182 days prior to the contralateral inoculation of $10^6$ tumour cells.

† Mean of 3 replicate samples.

‡ Days following tumour cell implantation.

§ Stimulation Index = ct/min of stimulated culture/ct/min of unstimulated culture (NaCl).

‖ Not Significantly different from control culture.

¶ Probability of significant difference from control culture (Student's *t* test).

(100 $\mu$g), and areas of cellular migration calculated.

The animals of this experiment were not sacrificed at the time of PE cell harvest, but followed for immunotherapeutic response to MER. The results (Table IV) indicated that 14 of 16 animals which had received MER, as opposed to none of 4 tumour-injected controls, showed immune responsiveness (*i.e.* $> 20\%$ inhibition of migration) against PPD (40 $\mu$g) ($P < 0{\cdot}001$; $\chi^2$ test). Reactivity to SA-10 (100 $\mu$g) was seen with 8 of 16 MER-treated, tumour-injected guinea-pigs and none of 4 Line-10-bearing controls ($P < 0{\cdot}01$; $\chi^2$ test). Moreover, borderline reactivity against SA-10 was detected in the case of 2 animals which had been inoculated only 3 days previously with tumour cells, a phenomenon possibly attributable to observed antigenic sharing between the Line 10 tumour and BCG (Minden *et al.*, 1974*a*). Only 3 of the 16 MER-treated animals of this experiment survived tumour challenge. The reasons for this relatively low survival rate are uncertain, but may be related to the fact that these animals had been traumatized both by stimulation and by the tapping of the peritoneal cavity for PE cells. Along these lines, it is worth noting that most of the animals of this experiment which succumbed, including non-MER-injected controls, died earlier (average day of death; 56 days) than did guinea-pigs which had not been subjected to experimental manipulation in the manner described (Table I, average day of death; 66 days).

### 4. *Lymphocyte stimulation*

The ability of SA-N and SA-10 to stimulate the peripheral lymphocytes of the MER-pretreated, tumour-injected guinea-pigs and tumour-bearing controls of Table I, Experiment I, was assessed by measuring incorporation of [$^3$H]thymidine, both in the presence and absence of stimulant. The results (Table V) showed specific reactivity to SA-10 to be present in each of 9 animals which had been inoculated 21 days previously with tumour cells. Levels of anti-SA-10 response in MER-pre-treated guinea-pigs were generally higher than those of controls, while reactivity against SA-N was not detected. As reported for the results of DCH studies, however, no differences were observed between MER-pretreated animals which survived tumour challenge and those which did not. These results supplement similar observations on levels of anti-tumour immunity in Line 10 tumour-bearing guinea-pigs which had been treated immunotherapeutically with living BCG (Littman *et al.*, 1973).

### DISCUSSION

The results reported here confirm previous observations from our laboratories that MER is an effective immunoprophylactic agent against the transplantable Line 10 hepatocarcinoma in Strain 2 guinea-pigs obtained from the breeding colonies of the Weizmann Institute of Science, Rehovot, Israel (Minden *et al.*, 1974*b*). Moreover, MER administration may precede tumour cell implantation by as much as 6 months and still be effective.

This protective effect, however, is sensitive to experimental manipulation, and may be abrogated by the use of several tumour implantation sites. This suggests that the defence mechanisms of these animals, even following pretreatment with MER, may be unable to cope with the presence of two simultaneously developing tumour nodules, and points to the complexity of the circumstances in which tumour regression may occur. Along these same lines, it should be noted that MER pretreatment is apparently successful in preventing a proportion of Strain 2 guinea-pigs bred at either the Weizmann Institute of Science or the National Jewish Hospital, Denver, Colo., from developing cancer, but is without effect when animals raised at the National Institutes of Health (NIH) (Zbar *et al.*, 1976) are employed. Similarly, we have shown in immunotherapy protocols that inoculation of MER (0·5–1·0 mg) into developing tumour nodules, 7 days after implantation of $10^6$ Line 10 cells, is more effective in guinea-pigs of either Weizmann Institute or Denver than NIH origin (Wainberg, Margolese and Weiss, 1976). This raises the question whether the various substrains of guinea-pigs used in this research are isogenic with respect to one another, and more importantly, whether they may differ from the transplantable Line 10 hepatoma in terms of histocompatibility antigen make-up. Indeed, should histocompatibility differences exist, then at least part of the tumour rejection phenomenon which both we (Minden, *et al.*, 1974*b*; Wainberg *et al.*, 1976) and others (Zbar and Tanaka, 1971; Zbar, Bernstein and Rapp, 1971) have observed may be due to homograft rather than tumour immunity. If this is the case, however, it is nonetheless obvious that the histocompatibility differences must be minor, since tumour inoculation in the absence of immunoprophylaxis or immunotherapy has been invariably fatal for the animals we have studied. For this reason, it is fair to speak of anti-tumour immunity in this system. These difficulties may be intrinsic to many or all tumour transplantation models.

Our results also indicate that animals pretreated with MER generally develop cellular anti-tumour immunity both more rapidly and at higher levels than do non-MER-treated, tumour-bearing controls, as measured by each of 3 different procedures. This applies to all animals which receive MER immunoprophylaxis, regardless of whether they eventually survive tumour challenge or succumb to progressive disease. Thus, we have not been able to prognosticate the outcome of MER pretreatment by our various *in vitro* and *in vivo* testing procedures. Similar findings were previously reported for this model, based on immunotherapy experiments using live BCG (Littman *et al.*, 1973). These data may suggest, therefore, that the T lymphoid cell population which we have been monitoring by these techniques is not of foremost importance as far as tumour rejection is concerned. Others (Evans and Alexander, 1972; Droller and Remington, 1975; Norbury and Fidler, 1975) have suggested that the macrophage is the most likely candidate for the role of critical effector cell in tumour immunology.

Nevertheless, the heightened levels of cell-mediated anti-tumour immunity which we have observed following MER pretreatment may be related, perhaps in a cooperative way, to ultimate survival. Earlier studies with other models, while showing that BCG can exert a tumour immunoprophylactic effect, did not provide ancillary data as to whether or not overall stimulation of specific anti-tumour immunity also occurred (Old, Clarke and Benacerraf, 1959; Lemonde *et al.*, 1971; Keller and Hess, 1972). Our findings indicate that such stimulation does take place.

Supported by grants from the Conseil de la Recherche en Santé du Québec, the Cancer Research Society, Inc., Montreal, and the National Cancer Institute of Canada, and by Contracts No. I-CB-02208 and No. I-CM-12127 from the National Cancer Institute, NIH.

### REFERENCES

BAST, R. C., JR., ZBAR, B., BORSOS, T. & RAPP, H. J. (1974) BCG and Cancer. *New Engl. J. Med.*, **290**, 1413 and 1458.

BEN-EFRAIM, S., CONSTANTINI-SOUROJON, M. & WEISS, D.W. (1973) Potentiation and Modulation of the Immune Response of Guinea-Pigs to Poorly Immunogenic Protein-Hapten Conjugates by Pretreatment with MER Fraction of Attenuated Tubercle Bacilli. *Cell Immunol.*, **7**, 370.

BLOOM, B. R. & BENNETT, B. (1971) The Assay of Inhibition of Macrophage Migration and the Production of Migration Inhibitory Factor (MIF) and Skin Reactive Factor (SRF) in the Guinea-Pig. In *In Vitro Methods in Cell Mediated Immunity*. Ed. B. R. Bloom and P. Slade. New York: Academic Press.

BOYUM, A. (1968) Separation of Leucocytes from Blood and Bone Marrow. *Scand. J. clin. Invest.*, **21**, 9 (Suppl. 97).

COHEN, D., YRON, I., HABER, M., ROBINSON, E & WEISS, D. W. (1975) Effect of Treatment with the MER Tubercle Bacilli Fraction on the Survival of Mice Carrying Mammary Tumour Isografts: Injection of MER at the Tumour Site or at a Distal Location. *Br. J. Cancer*, **32**, 483..

DROLLER, M. J. & REMINGTON, J. S. (1975) A Role for the Macrophage in *In Vivo* and *In Vitro* Resistance to Murine Bladder Tumour Cell Growth. *Cancer Res.*, **35**, 49.

EVANS, R. & ALEXANDER, P. (1972) Mechanism of Immunologically Specific Killing of Tumour Cells by Macrophages. *Nature, Lond.*, **236**, 168.

HARAN-GHERA, N. & WEISS, D. W. (1973) Effect of Treatment of C57 BL/6 Mice with the Methanol Extraction Residue Fraction of BCG on Leukemogenesis Induced by the Radiation Leukemia Virus. *J. natn. Cancer Inst.*, **50**, 229.

HOPPER, D. G., PIMM, M. V. & BALDWIN, R. W. (1975) Methanol Extraction Residue of BCG in the Treatment of Transplanted Rat Tumors. *Br. J. Cancer*, **31**, 176.

JOKIPII, L. & JOKIPII, A. M. M. (1974) Determination of the Migration Area in Macrophage Migration Inhibition Assay: A Comparison of the Whole with the Dense Part of the Fan. *J. Immunol. Methods*, **5**, 83.

KELLER, R. & HESS, M. W. (1972) Tumour Growth and Non-Specific Immunity in Rats: The Mechanisms Involved in Inhibition of Tumour Growth. *Br. J. exp. Path.*, **53**, 570.

KUPERMAN, O., YASHPHE, D. J., BEN-EFRAIM, S., SHARF, S. & WEISS, D. W. (1972) Nonspecific Stimulation of Cellular Immunological Responsiveness by a Mycobacterial Fraction. *Cell Immunol.*, **3**, 277.

LEMONDE, P., DUBREUIL, R., GUINDON, A. & LUSSIER, G. (1971) Stimulating Influence of Bacillus Calmette-Guerin on Immunity to Polyoma Tumours and Spontaneous Leukemia. *J. natn. Cancer Inst.*, **47**, 1013.

LITTMAN, B. H., MELTZER, M. S., CLEVELAND, R. P., ZBAR, B. & RAPP, H. J. (1973) Tumour-Specific, Cell-Mediated Immunity in Guinea-Pigs with Tumors. *J. natn. Cancer Inst.*, **51**, 1627.

LOWRY, O. H., ROSEBROUGH, N. J., FARR, A. L. & RANDALL, R. J. (1951) Protein Measurement with the Folin Phenol Reagent. *J. biol. Chem.*, **193**, 265.

MATHÉ, G., POUILLART, P. & LAPEYRAQUE, F. (1969) Active Immunotherapy of L1210 Leukemia Applied after the Graft of Tumor Cells. *Br. J. Cancer*, **23**, 814.

MELTZER, M. S., LEONARD, E. J., RAPP, H. J. & BORSOS, T. (1971) Tumor-Specific Antigen Solubilized by Hypertonic Potassium Chloride. *J. natn. Cancer Inst.*, **47**, 703.

MINDEN, P. & FARR, R. S. (1969) Binding Between Components of the Tubercle Bacillus and Humoral Antibodies. *J. exp. Med.*, **130**, 931.

MINDEN, P., MCCLATCHY, J. K., WAINBERG, M. & WEISS, D. W. (1974*a*) Shared Antigens Between Mycobacterium Bovis (BCG) and Neoplastic Cells. *J. natn. Cancer Inst.*, **53**, 1325.

MINDEN, P., WAINBERG, M. & WEISS, D. W. (1974*b*) Protection Against Guinea-Pig Hepatomas by Pre-treatment with Subcellular Fractions of Mycobacterium Bovis (BCG). *J. natn. Cancer Inst.*, **52**, 1643.

NORBURY, K. C. & FIDLER, I. J. (1975) *In Vitro* Tumor Cell Destruction by Syngeneic Mouse Macrophages: Methods for Assaying Cytotoxicity. *J. Immunol. Methods*, **7**, 109.

OLD, L. J., CLARKE, D. A. & BENACERRAF, B. (1959) Effect of Bacillus Calmette–Guerin Infection on Transplanted Tumours in the Mouse. *Nature, Lond.*, **184**, 291.

WAINBERG, M. A., MARGOLESE, R. G. & WEISS, D. W. (1976) Tumor Immunoprophylaxis and Immunotherapy in Guinea-Pigs Treated with the Methanol Extraction Residue (MER) of BCG. In *The Present Status of BCG in Cancer Immunotherapy*. Eds. G. Lamoureux, V. Portelance and R. Turcotte. New York: Grune & Stratton.

WEISS, D. W., STUPP, Y., MANY, N. & IZAK, G. (1975) Treatment of Acute Myelocytic Leukemia (AML) Patients with the MER Tubercle Bacillus Fraction. A Preliminary Report. *Transpl. Proc*,. **7**, 545 (Suppl. 1).

WEISS, D. W. & WELLS, A. W. (1960) Vaccination Against Tuberculosis with Non-Living Vaccines. III. Vaccination of Guinea-Pigs with Fractions of Phenol-Killed Tubercle Bacilli. *Am. Rev. resp. Dis.*, **82**, 339.

ZBAR, B., BERNSTEIN, I. D. & RAPP, H. J. (1971) Suppression of Tumor Growth at the Site of Infection with Living Bacillus Calmette–Guerin. *J. natn. Cancer Inst*,, **46**, 831.

ZBAR, B., MINDEN, P., MCCLATCHY, J. K. & RAPP, H. J. (1976) Prevention of Tumor Growth after Intradermal Injection of Extracts of BCG: Comparison of Results in Strain 2 Guinea-Pigs Obtained from the National Institutes of Health and from the National Jewish Hospital and Research Center. *J. natn. Cancer Inst.*, **56**, 443.

ZBAR, B. & TANAKA, T. (1971) Immunotherapy of Cancer: Regression of Tumor After Intralesional Injection of Living Mycobacterium Bovis. *Science, N.Y.*, **172**, 271.

ZBAR, B., WEPSIC, H. T., RAPP, H. J., WHANG-PENG, J. & BORSOS, T. (1969) Transplantable Hepatomas Induced in Strain 2 Guinea-Pigs by Diethylnitrosamine: Characterization by Histology, Growth and Chromosomes. *J. natn. Cancer Inst.*, **43**, 821.